# Automatic and manual image fusion of ^111^In-pentetreotide SPECT and diagnostic CT in neuroendocrine tumor imaging – An evaluation

**DOI:** 10.4103/0971-6203.71766

**Published:** 2010

**Authors:** Elisabeth Hedlund, Jan-Erik Karlsson, Sven-Åke Starck

**Affiliations:** 1 Medical Imaging, School of Health Sciences, Jönköping University, Jönköping, Sweden; 2 Department of Clinical Physiology, County Hospital Ryhov, Jönköping, Sweden; 3 Hospital Physics Unit, Department of Oncology, County Hospital Ryhov, Jönköping, Sweden

**Keywords:** Computed tomography, image fusion, neuroendocrine tumors, single photon emission computed tomography, somatostatin receptor scintigraphy

## Abstract

In the clinical diagnosis of neuroendocrine tumors (NET), the results of examinations, such as high-resolution computed tomography (CT) and single photon computerized tomography (SPECT), have conventionally been interpreted separately. The aim of the present study was to evaluate Hermes Multimodality™ 5.0 H Image Fusion software-based automatic and manual image fusion of SPECT and CT for the localization of NET lesions. Out of 34 NET patients who were examined by means of somatostatin receptor scintigraphy (SRS) with 111In- pentetreotide along with SPECT, 22 patients had a CT examination of the abdomen, which was used in the fusion analysis. SPECT and CT data were fused using software with a registration algorithm based on normalized mutual information. The criteria for acceptable fusion were established at a maximum cranial or caudal dislocation of 25 mm between the images and at a reasonable consensus (in order of less than 1 cm) between outline of the reference organs. The automatic fusion was acceptable in 13 of the 22 examinations, whereas 9 fusions were not. However all the 22 examinations were acceptable at the manual fusion. The result of automatic fusion was better when the slice thickness of 5 mm was applied at CT examination, when the number of slices was below 100 in CT data and when both examinations included uptakes of pathological lesions. Retrospective manual image fusion of SPECT and CT is a relatively inexpensive but reliable method to be used in NET imaging. Automatic image fusion with specified software of SPECT and CT acts better when the number of CT slices is reduced to the SPECT volume and when corresponding pathological lesions appear at both SPECT and CT examinations.

## Introduction

Somatostatin receptor scintigraphy (SRS) with ^111^In-pentetreotide, along with single photon emission computerized tomography (SPECT), plays an important role in neuroendocrine tumor (NET) imaging. The uptake can be highly tumor-specific and provides information on the receptor status. However, it can be impossible to localize the uptake anatomically. Nevertheless, the localization is usually facilitated by means of co-registration with computerized tomography (CT). An image fusion[1] is mainly used in order to combine images from CT and magnetic resonance imaging (MRI) with positron emission tomography (PET) and SPECT. The goal of an image fusion is to cover a structural anatomical framework on functional images. The reason is that in a functional image, there are often not enough of structural details to determine the anatomical position of an NET.[2] Traditionally, the results of either anatomical or functional pieces of information have been separately interpreted. In a multimodal environment in which allowance is made for both appearance and localization, the interpreter can make an evaluation along with both anatomical and physiological changes. To make a multimodal environment possible, an acceptable image fusion has to be produced.

In general, combining structural and functional images can be achieved prospectively with the use of stereotactic frames or external markers.[3] Retrospective techniques with manual co-registration or automatic methods based on surface or voxel intensity information[4–7] are shown to have an impact on patient management. Internal anatomical landmarks eliminate the need for external markers and patient preparation. Reliable identification and accurate localization of these landmarks is, however, not always possible, since it requires skillfulness and experience from the operator when automatic detection fails.[8]

In the NET diagnosis there is a lack of good reference points, because the normal distribution of ^111^In-pentetreotide entails uptake in only a few anatomical structures (e.g. liver, kidneys and spleen).[3] In addition, chest and abdomen are no rigid structures, and therefore differences in patient positioning and respiratory motion can make it difficult to align the anatomical and functional images.[7]

Gamma cameras with an integrated CT (SPECT/CT)[7,9] allow a precise interpretation of the results of scintigraphic studies with accurate localizations and a higher specificity in the diagnosis of NETs.[10–12] However, these equipments are still not in frequent use.

The aims of the present study were: (i) to analyze and evaluate both automatic and manual image fusion with Hermes MultiModality™ 5.0 H Image Fusion (Hermes Medical Solutions, Stockholm, Sweden) of retrospective examinations of SPECT and CT in NET imaging of the abdomen, (ii) to find out how many automatic and manual fusions were accepted and (iii) whether and if there were any elements affecting the different methods of fusion.

## Materials and Methods

During a three-year period that is, 2006, 2007 and 2008, 34 patients were investigated with SRS for NET, including SPECT. Out of the population, 22 patients (12 women, 10 men; age range 41–84 years; mean age ± SD, 65.9 ± 21,5 years) had an examination with CT abdomen and they were included in the study.

Image fusion was based on image data from the corresponding SPECT and CT examinations of each patient. Acquisition of SRS images included whole-body scans obtained 4, 24 and sometimes 48 h, after the intravenous injection of 170–220 MBq ^111^In- pentetreotide (‘OctreoScan’; Mallinckrodt, Petten, the Netherlands). SPECT of the abdominal area and/or thorax was performed at 24 h, executed with a double-head gamma camera Siemens E.CAM, (Siemens Medical Solutions, Hoffman Estates, Illinois, USA) with a medium energy collimator. Image acquisition was performed with a 128 × 128 matrix, 360 degree of rotation, 64 projections and 90 s/projection. Iterative reconstruction was made with six iterations, eight subsets, not filtered or post-filtered with a Butterworth filter, cut off 0.9/cm order 10 (Hermes Medical Solutions, Stockholm, Sweden). The position of the patient was supine with arms above the head.

‘CT abdomen’ examinations were performed at four radiological clinics with different equipments: Siemens sensation 64 and 16 slice (Siemens Medical Solutions, Forchheim, Germany), SOMATOM Emotion-16, 16-slice Siemens AG 2004, 16 slice CT, Siemens Medical Syngo CT 2006 G and Millennium VG and Hawkeye (GE Medical System, Chalfont St. Giles, United Kingdom). Scan parameters were 3 or 5 mm slice thickness, abdomen or thorax/abdomen. Reconstruction was made with body kernel (B30, B31, B40 or B41). CT scan was performed within an interval between 730 days before scintigraphy until 6 days after. The normal position of the patient was supine with arms above the head. The acquisition took approximately 20–25 s (depending on the patient’s length) and the patient held breath during the procedure.

SPECT and CT image data were transferred in DICOM format to Hermes GOLD 2.10 workstation via the PACS system. Registrations of CT and SPECT data were performed using Hermes MultiModality™ 5.0 H Image Fusion. The software adjusted the pixel size (4.8 mm), the number of cross-sections, the separation of slices and the orientation in the SPECT-study, so that the SPECT images were adapted to the CT images (pixel size 1–1.5 mm). To evaluate whether the image fusion was acceptable or not, the normal distribution of ^111^In-pentetreotide was studied in kidneys, liver, and spleen to examine whether it was equivalent to corresponding organs in the CT image.

The automatic fusion was built on maximization of mutual information based on informational claims and the method was proposed to be used for fusion of CT and SPECT.[13] The automatic fusion was accomplished on all the patients three times on different occasions.

Collected data were those of age, differences in time between collected CT and SPECT, CT protocol (slice thickness and number of CT slices), question formulation, pathological findings and information from physician’s referrals.

Table 1 presents the data about the examinations and the cohort of patients used in the study.

**Table 1:** Values for the cohort of patients at image fusion of SPECT and CT

*Difference in time (days) CT-SPECT*	*Exam area CT (slice thickness, mm)*	*CT number of slices*	*Pathology*
60	Thorax/abdomen (5.0)	81	0
22	Thorax/abdomen (3.0)	386	0
- 6	Abdomen (3.0)	324	0
36	Abdomen (5.0)	91	+
190	Abdomen (5.0)	71	0
48	Abdomen (superior) (5.0)	70	+
22	Thorax/abdomen (3.0)	416	0
5	Abdomen (5.0)	81	+
730	Abdomen (5.0)	72	+
-5	Abdomen (5.0)	91	+
136	Thorax/Abdomen (5.0)	96	+
3	Abdomen (5.0)	76	0
34	Abdomen (5.0)	89	+
0	Abdomen (5.0)	90	+
5	Thorax/abdomen (5.0)	90	+
12	Abdomen (5.0)	102	0
76	Abdomen (5.0)	74	+
36	Abdomen low dose (3.0)	223	0
49	Abdomen (5.0)	92	+
17	Abdomen (5.0)	76	0
52	Abdomen low dose (3.0)	282	+
168	Abdomen (5.0)	81	0

Manual fusion was based on landmarks selected by the operator. In both images, well-known landmarks were placed in corresponding coordinates at kidneys, liver and spleen. They could range in numbers and depended on the quality of images, visualized anatomy and radioactivity distribution. Collected data at manual fusion were number and average error between landmarks. The manual fusion was repeated for cases having an average error above 11 mm. The fusion was categorized into: (i) no dislocation, (ii) cranial or caudal dislocation and (iii) no concordance between SPECT and CT images.

The criteria determined for an acceptable image fusion were a maximum cranial or caudal dislocation of 25 mm, between the images, and a reasonable consensus (in order of less than 1 cm) between outline of the reference organs; liver, kidneys and spleen. The criterion 25 mm for dislocation was chosen because liver, spleen and kidneys are large structures and are subject to respiratory motion in the SPECT studies. Dislocation of fusion was subjectively estimated from the number of transverse slices being displaced between the images of SPECT and CT, with a slice thickness of 4.78 mm.

Arithmetic means and standard deviations were calculated for collected data.

## Results

As accounted for the above, in the study of image fusion of SPECT and CT, 22 examinations were analyzed, all of them with both automatic and manual fusion. Table 2presents the results. Applying to the criteria given above, the automatic image fusion was accepted in 13 out of the 22 examinations (59%), whereas 9 fusions were not (41%). However, all 22 examinations were accepted by means of manual fusion, including both test one and test two.

**Table 2:** Results of automatic and manual image fusion of SPECT and CT

*Automatic fusion*		*Manual fusion*		
*Acceptable fusion*	*Dislocation (mm)*	*Landmarks (number)*	*Test 1 average error (mm)*	*Test 2 average error (mm)*
Yes	10	5	11	11
No	47	7	11	12
No	No concordance	7	6
Yes	5-15	5	16	11
Yes	5	7	13	12
Yes	25	5	29	7
No	No concordance	7	9
Yes	No dislocation	7	11
Yes	20	7	16	12
Yes	16	7	8
Yes	No dislocation	7	12	10
No	50-60	7	10
Yes	15-20	7	14	11
No	No concordance	7	13	11
No	100	7	9
No	45-50	4	12	12
Yes	5-10	7	10
No	No concordance	4	8
Yes	5-10	5	28	12
Yes	15	7	17	12
Yes	No dislocation	6	5
No	No	7	13	12

Table 3shows mean values and standard deviations of the data in the study at the automatic fusion. A difference could be noted with regard to the number of CT slices. 12 out of 13 acceptable fusions had 82.0 ± 9.4 CT slices; one acceptable fusion had 282 slices. Thus, all acceptable fusions had less than 100 CT slices in the examination. A difference could also be noted with regard to the slice thickness at the CT examinations; when it was 5 mm, 12 examinations (71%) had an acceptable automatic fusion, whereas only 1 out of 5 (20%) examinations were acceptable when it was 3 mm. However, all examinations with 3 mm had more than 200 CT slices, so if the acceptance is dependent in the number of CT slices, the slice thickness could not be separated.

**Table 3:** Results of acceptable and not acceptable image fusion

	*Total 22 (100%)*	*Acceptable 13 (59%)*	*Not acceptable 9 (41%)*
Time difference (days)	77 ± 156 (-6-730)	110± 194 (-5-730)	29 ± 54 (-6-168)
Slice thickness (5.0 mm)	17 (77%)	12 (71%)	5 (29%)
Slice thickness (3.0 mm)	5 (23%)	1 (20%)	4 (80%)
Number of slices (CT)	138 ± 110 (70-416)	97 ± 56 (70-282)	199 ± 141 (76-416)
Pathologic uptake	12 (55%)	10 (83%)	2 (17%)
No pathologic uptake	10 (45%)	3 (30%)	7 (70%)

In order to find out whether the manual method could become reliable, a new test was carried out. Manual fusions with a mean error more than 11 mm were remade at test two. It resulted in a reduced average error from 12.8 to 10.1 mm.

**Figure 1:**
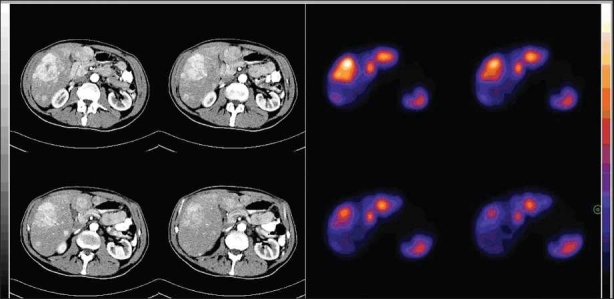
An image fusion in which positive uptake of activity in SPECT image (right) corresponds to NET lesions in the liver at CT image (left). There are also corresponding physiological uptakes in gall bladder and left kidney (left posterolateral aspect). Right kidney has no uptake in the SPECT study

**Figure 2:**
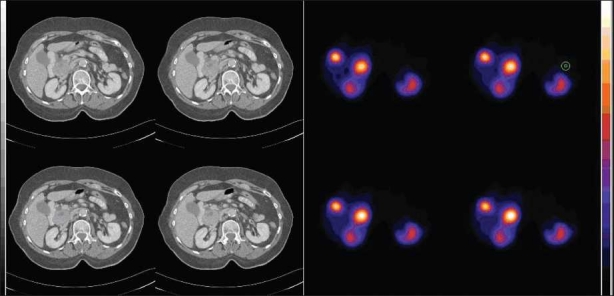
An example of image fusion where uptake of activity in SPECT image (right) can be localized to the pancreatic head (centre uptake) and physiological uptake in gall bladder (upper left uptake) and kidneys (inferior uptakes)

**Figure 3:**
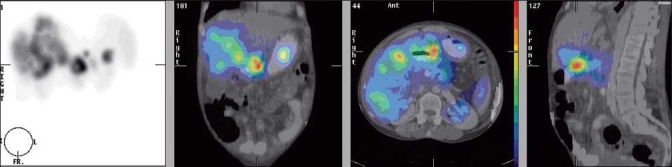
Fusion image of SPECT and CT. From left to right: SPECT MIP view, coronal view, transversal view and sagittal view. Positive uptake is located in liver metastases (diffuse uptake to the right), pancreas (high uptake in centre and in sagittal view) and stomach (left in coronal view)

An access to multimodal information data can of course; confirm or exclude NETs, from observations made giving anatomical explanations to some of the scintigraphic findings [Figure 1], thus reducing false interpretations of the uptake of activity. Typical examples are physiological tracer uptake in the gut or the gallbladder [Figure 2]. Figure 3 shows a result image of a fusion in three orthogonal planes with specific pentetreotide uptake in liver metastases and pancreas, and physiological uptake in the stomach.

## Discussion

As accounted for in the Introduction, the present study aims at an evaluation and an analysis of the software MultiModality™ 5.0, automatic and manual fusion methods of SPECT and CT, in the NET imaging. An automatic fusion is settled by the software itself but manual fusion requires the operator’s knowledge and experience for trustworthy placing of landmarks. In the present study, an automatic fusion was not always functioning, but the manual fusion was possible in all the 22 investigations. The advantage of the automatic fusion is that it is quick to perform, while the manual fusion requires more time, knowledge and precision of the operator. Manual fusion seems to be more difficult to use when a mass of neoplastic tissue hide structures being used as reference points, when the reference organs have been extirpated and when a part of reference organ is not fully visualized. It can also be additionally complicated with manual fusion when a pathological uptake of the tracer predominates over the visualized activity in the reference organs.

The range of the differences in time between CT and SPECT was from two years before, until six days after the scintigraphic examination. On an average, there were 110 days between the examinations, when automatic fusions of SPECT and CT were accepted, and on an average 29 days when automatic fusion was not accepted. One accepted automatic fusion took place between CT and SPECT when there was two years between the examinations, while one automatic fusion did not succeed despite that CT examination was settled six days after SPECT, on purpose to be evaluated with SPECT. In this case, the number of slices was 324 showing the importance of a special examination protocol when image fusion shall be made. To use CT material that is produced long before SPECT, there is a risk in anatomical changes like transposition or resection of organs. Automatic and manual fusion should actually work better, if there is less time between examinations, as no large organ transformation has taken place. Three patients in the study have undergone resection of the small intestine, part of the liver and nephrectomy, before the scintigraphy examination. The automatic fusion was not accepted for these patients.

Another variable that might influence the automatic fusion was the number of slices at CT examination. This is exemplified in Tables 1 and 3. Out of the six CT examinations with more than 100 slices, there was only one that worked by automatic fusion, 5 examinations did poorly or not at all. A possible explanation is that CT images outside the SPECT volume contained too extensive pieces of information, which disturbed the automatic fusion. Automatic fusion worked at 4 of 5 CT examinations, after manually reducing the number of slices, which fitted the region of the SPECT volume.

As shown in Table 3, the slice thickness at the CT examinations might have a potential impact on the acceptability of the automatic fusion. We have no good explanation for this observation. According to the method for CT examination of the abdomen, the investigator chooses slice thickness from actual clinical issue. A 5.0 mm slice thickness is chosen for imaging soft tissue and a 3.0 mm one for lesions in the skeleton. After all, a 5.0 mm thickness is more close to the slice thickness of 4.8 mm, conventionally used in SPECT of NETs.

An interesting observation was that that of all accepted automatic fusion, not less than 77% have pathological lesions. This fact might be another factor that could help automatic fusion to work more successfully. Obviously, the software seems to identify pathological structures in SPECT and CT examinations.

One approach to improve the automatic fusion could be to adjust the CT data, so that it better match with the SPECT data. This could be done, either by modifying CT data in the fusion program or by cooperation with the radiological department, to compose a suitable protocol for abdominal CT, for the purpose of fusion with SPECT. Slomka[5] claimed some years ago that compatibility between different modalities and cooperation between various clinical departments is of decisive importance for a successful application of software-based image fusion in a hospital setting. Here, an additional interesting study would be both to consider which examination protocol of abdominal CT could ultimately work in fusion with SPECT and to elaborate data for a method of description for a multimodal image fusion. Also of importance is the positioning of the patient on the scanner, for example arms above the head or beside the body.

The task to make a manual fusion a reliable identification and accurate localization of landmarks requires both skillfulness and experience from the operator.[8] Our observations are reported in Tables 2 and 3.

To the best of our knowledge, only a few scientific studies have more recently investigated which criteria should be established, in order to accept retrospective image fusion of SPECT and CT. When studying image fusion with various techniques Amthauer *et al*,[14,15] a few years ago used a subjective visual plausibility control of the shape and the physiological uptake of activity in the reference organs, such as the liver, the spleen and the kidneys. Their study[14] included 38 patients and they used an automatic method of fusion with voxel-based technique, built on normalized information. Two fusions are excluded, since they are not estimated as plausible. By two observers, SRS allowed a definite anatomical assignment in 57 and 61% of all lesions. Image fusion improved these figures to 91 and 93% by the two observers, respectively. Also image fusion improved assignment to the corresponding liver segment from 45 to 98% and from 58 to 100% by the two observers.

The quality control of image fusion used in the present study is subjected to certain limitations. In the study we used a determination of criteria for acceptable image fusion as a maximum cranial or caudal dislocation of 25 mm between the images and a reasonable consensus between outline of the three reference organs. It is also important not to level intensity in the SPECT images to much so it changes the size of an organ. The evaluation is subjective. Actually a visual assessment determines the number of slices that separates organs between SPECT and CT. Furthermore, quality control demands training for reliable judgment. Such a subjective assessment of fusion can differ considerably between diverse operators, since they presumably select different landmarks at the manual fusion.

In the present study, we depended on available examinations of abdominal CT, which were available to us. It should, therefore, be emphasized that the results could have been different with other kind of examinations than in just NET imaging, perhaps with more successful automatic fusions. The results show, however, that manual fusion always is an alternative when automatic fusion is unfeasible. Results from the study by Amthauer *et al*,[15] also illustrate that accurate anatomical localization of SRS foci in parenchymal organs were equally well suited between a SPECT/CT hybrid (low-dose X-ray tube) (overall accuracy 91%) and retrospective image fusion by means of software (94%).

## Conclusion

A gamma camera with an integrated diagnostic CT, combining transmission and emission tomography in the same session, is not a prevalent clinical reality yet. Retrospective manual image fusion of SPECT and CT is a relatively inexpensive but reliable method to use when dealing with NET. However, a manual fusion requires skillfulness and experience from the operator. Automatic image fusion with specified software of SPECT and CT functions better when the number of CT slices in CT is reduced to the SPECT volume and when the pathologic lesions appear in both SPECT and CT examinations.
